# Physicians’ irrational attitudes on the antibiotic prescribing for the treatment of COVID-19 in Turkey: A multicenter survey

**DOI:** 10.1186/s12913-024-11110-z

**Published:** 2024-05-21

**Authors:** Demet Polat Yuluğ, Berker Öztürk, Oya Baydar Toprak, Ebru Öztürk, Nurdan Köktürk, Sibel Naycı

**Affiliations:** 1 Department of Chest Diseases, Mersin City Training and Research Hospital, Mersin, Turkey; 2 Clinic of Chest Diseases, Private Cappadocia Hospital, Nevşehir, Turkey; 3 Department of Chest Diseases, Faculty of Medicine, Cukurova University, Adana, Turkey; 4 Department of Biostatistics, Hacettepe University, Ankara, Turkey; 5 Department of Chest Diseases, Faculty of Medicine, Gazi University, Ankara, Turkey; 6 Department of Chest Diseases, Faculty of Medicine, Mersin University, Mersin, Turkey

**Keywords:** Antibiotic stewardship, Physicians, Covid-19

## Abstract

**Background:**

The inappropriate and excessive use of antibiotics during the coronavirus pandemic has become an important issue.

**Objective:**

Our primary aim is to ascertain the attitudes of physicians toward the antibiotics prescribing for the treatment of COVID-19 in Turkey. Our secondary aim was to identify factors affecting to physicians’ decisions regarding antibiotic therapy for the treatment of COVID-19 and risk factors associated with antibiotic overprescribing.

**Methods:**

It was a multicenter cross-sectional survey. Physicians from 63 different cities were invited to survey through social media (Facebook, Instagram, WhatsApp). Data were collected from respondents through an online questionnaires during November-December 2021.

**Results:**

The survey was completed by 571 participants from 63 cities. Pulmonologists comprised the majority (35.20%), followed by internal medical specialists (27.85%) and general practitioners (23.29%). The rates of participants who started empirical antibiotics in the outpatient, ward, and ICU (intensive care unit) were 70.2%, 85.5%, and 74.6%, respectively. When the practice of prescribing antibiotics by physicians for the treatment of COVID-19 in outpatients was compared according to the healthcare setting (primary, secondary, tertiary care hospitals) no significant difference was found. Sputum purulence (68.2%) was recognized as the most important factor for the decision of antibiotic therapy, followed by procalcitonin levels (64.9%) and abnormal radiological findings (50.3%). The most prescribed antibiotics were respiratory quinolones. (48%, 65.9%, 62.7% outpatient, ward, ICU respectively)

**Conclusions:**

In this study, we found that physicians frequently had irrational attitudes toward antibiotic prescription to COVID-19 patients, including those with minor diseases. Our findings underline that the necessity of particular, workable interventions to guarantee the prudent use of antibiotics in COVID-19.

**Supplementary Information:**

The online version contains supplementary material available at 10.1186/s12913-024-11110-z.

## Introduction

The COVID-19 (coronavirus disease 2019) pandemic has crucially influenced antibiotic stewardship and increased the antibiotic use worldwide. Previous studies have shown that the frequency of secondary bacterial infections in COVID-19 patients is relatively low (overall population: 8–16%, critically ill patients: 16–31%) [1, 2]. However, some studies suggest an increase in inappropriate antibiotic prescribing practices for the treatment of COVID-19 (46-86%) [3–5]. In COVID-19, as in other viral and bacterial infections, there may be an increase in inflammatory markers and abnormal radiological images. Therefore, it is often difficult to distinguish COVID-19 from a bacterial infection [6]. Thus, there is potential for substantial overuse or inappropriate use of antibiotics in the management of COVID-19. This issue is important because inappropriate use of antibiotics causes significant problems such as the risk of antibiotic-related side effects, the risk of antimicrobial resistance and increased economic burden.

As of the date of this report (April 15, 2023), a total of 17.232.066 COVID-19 patients were detected in Turkey, resulting in 102.174 deaths according to data from the Turkish Ministry of Health [7]. In a multicenter study conducted in Turkey, the rate of antibiotic use in COVID-19 patients was 46% [5].

Although a few studies have reported the frequency of antibiotic prescribing for treatment of the COVID-19, there is a lack of knowledge regarding antibiotic prescribing attitudes and practices by physicians during the pandemic. Moreover, we still do not have any information on the antibiotic treatment practices by doctors treating COVID-19 patients in Turkey. In the current study, our hypothesis is that antibiotics are prescribed inappropriately in Turkey, even in patients with proven viral infections such as COVID-19. Our primary aim is to investigate antibiotic prescribing practices for the treatment of COVID-19. Our secondary aim is to identify factors affecting to physicians’ decisions regarding antibiotic therapy for the treatment of COVID-19 and risk factors associated with antibiotic overprescribing.

## Methods

### Ethical approval

This study was approved by the local institutional ethics committee of the Cukurova faculty, Adana, Turkey (approval No. 2021/116). This study was conducted in accordance with the hospital’s ethical standards, the national research committee and the 1975 Helsinki declaration.

### Study design

It was a multicenter, cross-sectional survey design study. Participation in the survey was voluntary. Before participating in the questionnaire, participants were informed about the aim of the study. Informed consent was obtained from all respondents before the questionnaire.

### Populations

Participants from 63 different cities were invited to survey through social media (Facebook, Instagram, WhatsApp). Each participant was included in the study according to the following criteria: (1) medical doctors and (2) involvement in the treatment of COVID-19 patients. The data were recorded between November and December 2021.

### Questionnaire

Before starting the survey, there was a little information about the study and informed consent. The main aim of this study is to represent the attitudes of clinicians toward prescribing antibiotics during the COVID-19 pandemic. The questionnaire (https://docs.google.com/forms/d/1DHNWAx_zmjc5Pa2pxkpXJ4k6nUXHWAZ0Vl-YkcNH6KQ/edit?%20ts=61a0a698, Supplement S1) used in this study was developed after searching the literature for similar studies [8, 9]. The questions in the survey consists of single or multiple-choice questions. The survey was completed, then two independent physicians who are studying at the Department of Chest Diseases for at least 15 years evaluated the survey. Thus, the corrections based on their suggestions were made. The survey consisted of 21 questions. The survey is composed of questions about demographic information, type of specialist, work experience duration, profession title and type of healthcare setting. They were asked what percentage (0–20%, 20–40%, 40–60%, 60–80%, 80–100%) of COVID-19 patients were prescribed antibiotics in the outpatient, ward and ICU settings. Overprescription was defined as more than 20%, 40% and 60% for outpatient, ward and ICU patients, respectively, considering co-infection prevalences in previous studies [1, 5]. According to the World Health Organization (WHO) definition, medicines are used rationally when patients receive the appropriate medicines, for appropriate indications, in doses that meet their own individual requirements, for an adequate period of time, at the lowest cost both to them and the society, and with appropriate information. Irrational or unnecessary use of medicines occurs when one or more of these conditions is not met [10]. Considering both the World Health Organization’s definition and the criteria for starting antibiotics in COVID-19 patients in the national COVID-19 guideline irrational antibiotic use was defined as starting antibiotics without evidence of radiological (lobar pneumonia, etc.), microbiological (sputum gram stain, culture result, etc.) or laboratory (procalcitonin) findings for bacterial infections [10, 11].

There were also questions about the most common antibiotic options prescribed for the treatment of COVID-19 patients. In addition, it was asked which of the clinical, laboratory and radiological variables was taken into account in deciding whether to start antibiotic therapy.

There were no open-ended questions. A restriction was set in the online survey link that an IP could only be used to fill out questionnaire once to avoid repeating questionnaire. We performed logic check and corrected and clean any non-logical data. The datasets of the study are available from the corresponding author upon reasonable request.

### Statistical analysis

The descriptive statistics were expressed as the mean and standard deviation for continuous variables, while frequency and percentages were given for categorical variables. Pearson’s chi-squared test or Fisher’s exact test was used to examine the association among two independent categorical variables. Logistic regression analysis was performed to determine factors affecting antibiotic overprescription (overprescription was defined as more than 20%, 40% and 60% for outpatient, ward and ICU patients, respectively [1, 5]). The candidate variables were chosen by univariate logistic regression models with a significance of *p* ≤ 0.25 to identify the factor for antibiotic prescribing. Backward elimination was used to find the final model using a multiple logistic regression model with those candidate variables. The level of statistical significance was considered 0.05. IBM SPSS version 23 was used for statistical analysis.

## Results

### Demographic characteristics

The survey was responded by 571 participants from 63 cities. Out of the 571 respondents, 317 (55.5%) were females. Pulmonologists comprised the majority (35.20%), followed by internal medical specialists (infection diseases, intensive care, and internal medicine) (27.85%), general practitioners (23.29%) and others (13.66%). 52.89% of the physicians were specialists, 20.14% were research assistants and 15.41% were lecturers. Physicians working in tertiary care hospitals comprised 59.72% of the study population. The majority (74.88%) of the physicians had 5 years or more of work experience. (Table 1)

**Table 1:** Demographic characteristics of the study physicians

Variable	Category	Descriptive Statistics *n* (%)
Gender	Female	317 (55.52)
	Male	254 (44.48)
Age (mean ± SD*)		37.03 ± 9.20
Profession	General practitioners	133 (23.29)
	Internal medical specialists (infection diseases, intensive care, and internal medicine specialists)	159 (27.85)
	Pulmonologists	201 (35.20)
	Others	78 (13.66)
Experience	1–5 years	144 (25.22)
	5–15 years	262 (45.88)
	15–25 years	103 (18.04)
	> 25 years	62 (10.86)
Type of Healthcare Setting	Primary	35 (6.13)
	Secondary	195 (34.15)
	Tertiary	341 (59.72)
Region^†^	Istanbul (TR1)	42 (7.36)
	West Marmara (TR2)	7 (1.23)
	Aegean (TR3)	42 (7.36)
	East Marmara (TR4)	46 (8.06)
	West Anatolia (TR5)	115 (20.14)
	Mediterranean (TR6)	100 (17.51)
	Central Anatolia (TR7)	99 (17.34)
	West Black Sea (TR8)	38 (6.65)
	East Black Sea (TR9)	17 (2.98)
	Northeast Anatolia (TRA)	15 (2.63)
	Central East Anatolia (TRB)	27 (4.73)
	Southeast Anatolia (TRC)	23 (4.03)

*SD: Standard deviation, ^†^Ü. Şengül, S. Eslemıan, M. Eren Türkiye’de İstatistikî Bölge Birimleri Sınıflamasına Göre Düzey 2 Bölgelerinin Ekonomik Etkinliklerinin VZA Yöntemi ile Belirlenmesi ve Tobit Model Uygulaması, Yönetim Bilimleri Dergisi, 11:21;75–99, 2013 (in Turkish)

### Antibiotic prescribing attitudes of participants

The rates of participants who started empirical antibiotics in the outpatient, ward, and intensive care units were 70.2%, 85.5%, and 74.6%, respectively. A total of 26.9% of physicians stated that they prefer to prescribe antibiotics for more than 40% of COVID-19 patients in outpatient clinics. When the practice of prescribing antibiotics by physicians for the treatment of COVID-19 in outpatients was compared according to the healthcare setting (primary, secondary, tertiary care hospitals) no significant difference was found.

A total of 61.8% of physicians stated that they prefer to prescribe antibiotics for more than 40% of hospitalized patients. While 43.81% of physicians prescribed antibiotics to almost all patients in secondary care hospitals for hospitalized COVID-19 patients, this rate was 19.4% in tertiary care hospitals and the difference was statistically significant. (*p* < 0.001)

A total of 85.6% of physicians stated that they prefer to prescribe antibiotics for more than 40% of patients in the ICU. Half of the physicians (51.2%) were prescribing antibiotics to almost all COVID-19 patients. While 67.19% of physicians prescribed antibiotics to almost all (80–100%) patients in secondary care hospitals for COVID-19 patients treated in the ICU, this rate was 44.37% in tertiary care hospitals, which was statistically significant. (*p* < 0.032)

The antibiotic prescription rates of the physicians were compared according to the years of work experience by dividing them into four groups (1–5, 5–15, 15–25, > 25 years). There was no significant difference between the groups. When the frequency of antibiotic prescribing for the treatment of COVID-19 patients followed in wards and ICU was compared according to the specialty of the participants, no significant difference was detected. For outpatients, 14.29% of pulmonologists and 5.63% of internal medicine specialists stated that they prescribe antibiotics to all outpatients. This rate was significantly higher than that of general practitioners. (p:0.032) Table 2 illustrate the frequency of antibiotic prescribing practices based on the profession.

**Table 2:** Frequency of prescribing antibiotics for treatment of COVID-19 patients based on duty ward and profession title of the participants

	Research assistant	Lecturer†	General Practitioner	Specialist Physician††	Test Statistic	*p*-value
**Outpatient**					NA	0.419
%0–20	43 (61.43)	31 (65.96)	20 (44.44)	92 (51.98)		
%21–40	13 (18.57)	9 (19.15)	11 (24.44)	30 (16.95)		
%41–60	7 (10)	5 (10.64)	10 (22.22)	28 (15.82)		
%61–80	4 (5.71)	1 (2.13)	1 (2.22)	16 (9.04)		
%81–100	3 (4.29)	1 (2.13)	3 (6.67)	11 (6.21)		
**Inpatient**					NA	**< 0.01**
%0–20	12 (19.35) ^a, b^	21 (30.88) ^b^	0 (0) ^a, b^	24 (14.12) ^a^		
%21–40	13 (20.97) ^a^	18 (26.47) ^a^	1 (14.29) ^a^	28 (16.47) ^a^		
%41–60	8 (12.9) ^a, b^	17 (25) ^a^	2 (28.57) ^a, b^	19 (11.18) ^a^		
%61–80	15 (24.19) ^a^	9 (13.24) ^a^	2 (28.57) ^a^	32 (18.82) ^a^		
%81–100	14 (22.58) ^a^	3 (4.41) ^b^	2 (28.57) ^a, b^	67 (39.41) ^a^		
ICU*					NA	**0.005**
%0–20	0 (0) ^a^	2 (4.65) ^a^	0 (0) ^a^	10 (8.33) ^a^		
%21–40	4 (10) ^a^	6 (13.95) ^a^	0 (0) ^a^	7 (5.83) ^a^		
%41–60	5 (12.5) ^a^	9 (20.93) ^a^	0 (0) ^a^	12 (10) ^a^		
%61–80	15 (37.5) ^a^	12 (27.91)^a, b^	1 (33.33) ^a, b^	17 (14.17) ^b^		
%81–100	16 (40) ^a, b^	14 (32.56) ^b^	2 (66.67) ^a, b^	74 (61.67) ^a^		

* ICU: Intensive care unit. Different letters represent statistically significant differences in column proportions. †Lecturer: physicians who care for patients at the university. ††Specialists: physicians working in hospitals other than university hospitals

### Factors affecting to physicians’ decisions regarding antibiotic therapy for the treatment of COVID-19

**Fig. 1:**
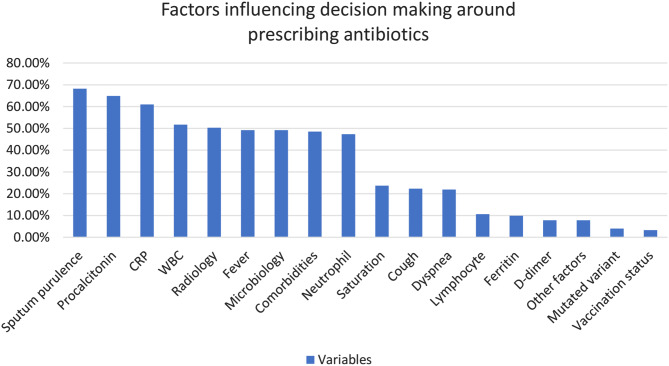
Factors influencing attitudes around prescribing antibiotics for the treatment of COVID-19 patients in the overall physician cohort

Sputum purulence (68.2%) was detected as the most common reason for the prescribtion of antibiotics, followed by laboratory markers and abnormal radiology findings (50.3%). The most important laboratory markers were procalcitonin (64.9%), followed by CRP (61%), WBC count (51.7%) and neutrophil count (47.3%) Other responses are listed in Fig. 1.

Univariable regression analysis to identify factors associated with the overprescribing antibiotics for the treatment of COVID-19 patients in the outpatient, ward and ICU setting were given in Table 3.

Fever, comorbidities and D-dimer were found to be significant among the factors affecting the antibiotic prescribing decision in the multiple logistic regression analysis for outpatients. (Table 4) The *Hosmer-Lemeshow* test of goodness of fit indicates that the model fits the data well (χ^2^ = 1.387, *p* = 0.846). The area under the ROC curves was found to be 0.709 (0.654–0.764) [12].

**Table 3:** Univariable regression analysis to identify factors associated with the overprescribing antibiotics for the treatment of COVID-19 patients in the outpatient, ward and ICU setting

Variables	Outpatient		Ward		ICU	
	OR (95% CI)	*p*-value	OR (95% CI)	*p*-value	OR (95% CI)	*p*-value
Age	1.006 (0.984–1.028)	0.603	0.994 (0.97–1.018)	0.603	0.993 (0.959–1.029)	0.697
Gender (Female)	0.785 (0.511–1.206)	0.269	0.847 (0.531–1.352)	0.487	0.863 (0.458–1.625)	0.648
Fever	3.653 (2.322–5.748)	< 0.001	3 (1.853–4.856)	< 0.001	2.201 (1.169–4.143)	0.015
Cough	3.168 (1.86–5.397)	< 0.001	4.642 (2.192–9.831)	< 0.001	4.752 (1.615–13.98)	0.005
Sputum purulence	1.113 (0.707–1.755)	0.643	0.978 (0.596–1.604)	0.93	1.243 (0.645–2.396)	0.515
Dyspnea	2.707 (1.61–4.551)	< 0.001	3.724 (1.745–7.946)	0.001	6.679 (1.978–22.555)	0.002
Comorbidities	2.032 (1.316–3.139)	0.001	2.248 (1.403–3.601)	0.001	3.625 (1.845–7.123)	< 0.001
Mutated variant	2.51 (0.741–8.502)	0.139	1.569 (0.481–5.124)	0.455	4.243 (0.535–33.659)	0.171
Saturation	1.972 (1.194–3.258)	0.008	2.782 (1.53–5.056)	0.001	2.911 (1.224–6.923)	0.016
Vaccination status	4.266 (1.153–15.788)	0.03	0.482 (0.127–1.831)	0.284	0.351 (0.069–1.795)	0.209
CRP	2.537 (1.599–4.024)	< 0.001	3.529 (2.158–5.771)	< 0.001	6.53 (3.325–12.826)	< 0.001
Procalcitonin	0.886 (0.571–1.373)	0.587	0.348 (0.176–0.688)	0.002	1.211 (0.518–2.834)	0.659
D-dimer	4.773 (1.874–12.153)	0.001	6.389 (1.46–27.956)	0.014	3.362 (0.751–15.056)	0.113
WBC	2.065 (1.336–3.194)	0.001	1.584 (0.996–2.518)	0.052	2.81 (1.491–5.298)	0.001
**Health care setting**						
Secondary care hospitals	1.225 (0.502–2.986)	0.656	-	-		
Tertiary care hospitals	0.975 (0.406–2.339)	0.955	0.56 (0.338–0.927)	0.024	0.607 (0.299–1.231)	0.167
**Work Experience**						
5–15 years	0.814 (0.481–1.378)	0.444	1.138 (0.562–2.306)	0.719	0.756 (0.302–1.893)	0.551
15–25 years	1.393 (0.725–2.676)	0.32	0.932 (0.423–2.058)	0.862	0.745 (0.228–2.437)	0.626
> 25 years	0.745 (0.357–1.555)	0.433	1.413 (0.769–2.596)	0.265	1.091 (0.476–2.502)	0.837
**Profession title of the physicians**						
Lecturer†	0.822 (0.38–1.778)	0.619	0.45 (0.227–0.891)	0.022	0.405 (0.156–1.053)	0.064
General Practitioner	1.991 (0.931–4.256)	0.076	-	-	-	-
Specialist††	1.471 (0.837–2.587)	0.18	1.372 (0.764–2.466)	0.29	0.831 (0.357–1.934)	0.667

CRP: C-reactive protein, WBC: White blood cell, OR: Odds ratio, CI: Confidence interval †Lecturer: physicians who care for patients at the university. ††Specialists: physicians working in hospitals other than university hospitals

**Table 4:** Multivariable regression analysis to identify factors associated with the overprescribing antibiotics for the treatment of COVID-19 patients in the outpatient, ward and ICU setting

Variables	Outpatient		Ward		ICU	
	OR (95% CI)	*p*-value	OR (95% CI)	*p*-value	OR (95% CI)	*p*-value
Fever	3.189 (2.004–5.076)	< 0.001	-	-	-	-
Cough	-	-	2.643 (1.181–5.916)	0.018	-	-
Dyspnea	-	-	-	-	3.637 (1.01–13.091)	0.048
Comorbidities	1.801 (1.135–2.857)	0.012	1.863 (1.1–3.155)	0.021	2.682 (1.285–5.601)	0.009
CRP	-	-	3.128 (1.793–5.458)	< 0.001	5.156 (2.551–10.425)	< 0.001
Procalcitonin	-	-	0.29 (0.137–0.613)	0.001	-	-
D-dimer	3.823 (1.439–10.154)	0.007	4.429 (0.924–21.221)	0.063	-	-
**Profession title of the physicians**						
Lecturer†	-	-	0.512 (0.239–1.099)	0.086	-	-
Specialist††	-	-	1.445 (0.761–2.741)	0.26	-	-

CRP: C-reactive protein, WBC: White blood cell, OR: Odds ratio, CI: Confidence interval. †Lecturer: physicians who care for patients at the university. ††Specialists: physicians working in hospitals other than university hospitals

Cough, comorbidities, CRP, D-dimer and being a specialist physician were found to be independently and positively associated with prescribing antibiotics for hospitalized patients. Procalcitonin and being a lecturer were associated with lower odds of receiving an antibiotic. (Table 4) The *Hosmer-Lemeshow* test of goodness of fit indicates that the model fits the data well (χ^2^ = 12.893, *p* = 0.116). The area under the ROC curve was found to be 0.767 (0.713–0.821) with a 95% confidence interval for predicted probabilities, indicating acceptable discrimination [12].

Dyspnea, comorbidities and CRP were found to be significantly and positively associated with antibiotic prescribing decisions for the patients in the ICU in the multiple logistic regression analysis. (Table 4) The *Hosmer-Lemeshow* test of goodness of fit indicates that the model fits the data well (χ^2^ = 4.909, *p* = 0.427). The area under the ROC curve was found to be 0.782 (0.712–0.853) with a 95% confidence interval for predicted probabilities, indicating acceptable discrimination [12].

### Antibiotic prescribing practices

Quinolones were the most preferred antibiotics for COVID-19 in outpatients (48%) and inpatients (65.9%) as well as in the ICU (62.7%). Table 5 shows the practice of physicians in prescribing antibiotics for the treatment of patients with COVID-19. When the types of antibiotics prescribed according to the type of health care setting were compared, no statistically significant difference was found.

**Table 5:** The practice of physicians in prescribing antibiotics

	General Practitioners	Internal medical specialists†	Pulmonologists	Others	Test Statistic	*p*-value
**Outpatient**						
Cephalosporins	16 (12.03) ^a^	9 (5.66) ^a, b^	16 (7.96) ^a, b^	1 (1.28) ^b^	9.265	**0.026**
Fluoroquinolone	38 (28.57) ^a, b^	39 (24.53) ^b, c^	76 (37.81) ^a^	9 (11.54) ^c^	20.847	**< 0.001**
Macrolide	29 (21.8) ^a^	12 (7.55) ^b^	41 (20.4) ^a^	5 (6.41) ^b^	20.573	**< 0.001**
Penicillin / amoxicillin	15 (11.28)	11 (6.92)	15 (7.46)	4 (5.13)	3.18	0.365
Combination	5 (3.76)	8 (5.03)	5 (2.49)	2 (2.56)	NA	0.617
**Inpatient**						
Cephalosporins	14 (56)	40 (42.55)	90 (54.88)	12 (50)	3.926	0.27
Fluoroquinolone	12 (48)	61 (64.89)	117 (71.34)	11 (45.83)	9.984	**0.019**
Beta lactam + Beta lactamase inhibitor	7 (28)	19 (20.21)	43 (26.22)	1 (4.17)	6.565	0.087
Antipseudomonal beta lactam	2 (8) ^a, b^	26 (27.66) ^b^	24 (14.63) ^a, b^	0 (0) ^b^	NA	**0.002**
Carbapenem	4 (16)	17 (18.09)	14 (8.54)	3 (12.5)	NA	0.128
Glycopeptide	0 (0)	3 (3.19)	2 (1.22)	1 (4.17)	NA	0.418
Macrolide	7 (28)	22 (23.4)	37 (22.56)	5 (20.83)	0.439	0.929
**ICU**						
Cephalosporins	6 (46.15)	18 (29.51)	27 (29.35)	15 (37.5)	2.223	0.527
Fluoroquinolone	6 (46.15)	37 (60.66)	63 (68.48)	22 (55)	3.907	0.272
Beta lactam + Beta lactamase inhibitor	3 (23.08)	23 (37.7)	30 (32.61)	6 (15)	6.592	0.086
Antipseudomonal beta lactam	2 (15.38)	34 (55.74)	59 (64.13)	6 (15)	33.197	**< 0.001**
Carbapenem	7 (53.85)	41 (67.21)	52 (56.52)	14 (35)	10.239	**0.017**
Glycopeptide	2 (15.38)	14 (22.95)	15 (16.3)	3 (7.5)	4.207	0.24
Macrolide	3 (23.08)	19 (31.15)	15 (16.3)	9 (22.5)	4.665	0.198

†Infection diseases, internal medicine and intensive care specialists. NA: Not applicable ICU: Intensive care unit. Different letters represent statistically significant differences in column proportions

## Discussion

In our current study, we found that irrational antibiotic prescribing practices to treat COVID-19 patients are common among physicians. The rates of participants who started empirical antibiotics in the outpatient, ward, and intensive care units were 70.2%, 85.5%, and 74.6%, respectively. Sputum purulence (68.2%) was detected as the most common reason for the prescription of antibiotics, followed by laboratory markers and abnormal radiology findings (50.3%). The most prescribed antibiotics were respiratory quinolones. To our knowledge, this is the first survey to investigate the antibiotic prescribing attitudes of physicians and the factors that affect their decision to prescribe antibiotics for the treatment of COVID-19 patients in Turkey.

In a large-scale study conducted mainly with hospitalized Covid-19 patients in Turkey, the rate of antibiotic use was 46%, and antibiotic use was associated with a 9.29-fold increase in mortality [5]. Another a multi-center study from Turkey was reported that two-thirds of the patients hospitalized with a diagnosis of COVID-19 received antibiotics and the inappropriate antibiotic prescribing rate was 71.2% [13]. However, only hospitalized patients were included in this study. Although there are methodological differences between this study and our study, our findings are consistent with the results of this study. A survey study conducted in 23 countries, including Turkey, showed that only 29.1% of participants chose not to prescribe an antibiotic for hospitalized patients. However, that study was conducted with a limited number of participants [9]. In another survey of 511 physicians, antibiotic prescribing practices varied between 72% and 87% according to the severity of patients’ illness. However, in that study, there were no data on the specialties of physicians [14]. In the present study, similar to previous studies, a high rate of prescribing antibiotics to COVID-19 patients was found among physicians. In addition, we collected more detailed information about attitudes toward prescribing antibotics for the treatment of COVID-19 such as profession, type of healthcare setting and work experience. We found that more antibiotics were prescribed in secondary care hospitals than in tertiary hospitals, especially in ward and intensive care patients. The high rate of antibiotic prescriptions for COVID-19 patients who our investigation discovered was against both the national and international recommendations for COVID-19 treatment [11, 15]. Antibiotic resistance may have increased as a result of inappropriate and illogical usage of antibiotics, especially during the COVID-19 pandemic.

The presented data show that the decision on antibiotic use was based on the presence of sputum purulance followed by high procalsitonin, CRP, WBC and abnormal radiology. Physicians believed that procalcitonin was the most significant test parameter to affecting antibiotic prescribing. This finding is consistent with the results of multicenter studies previously conducted in 23 countries previously [9]. There may be an increase in procalcitonin levels due to bacterial coinfections and lung damage due to cytokine release. Martins-Filo et al. showed that procalcitonin levels were associated with the severity of the disease in the COVID-19 pandemic [16]. Importantly, it was shown that the frequency of coinfection was only 20% and 50% in severe and critically ill COVID-19 patients, while elevated procalcitonin levels were 50% and 80%, respectively [17, 18]. As a result, we believe that choosing antibiotics based on procalcitone should be done with caution.

Almost half of the physicians (49.20%) thought that if COVID-19 patients had fever, antibiotics should be given. This finding is consistent with previous study, which stated that more than half of physicians considered high fever when reporting their antibiotic prescribing practices [19].

The study that was presented demonstrated that CRP was a significant factor among those influencing the decision to prescribe antibiotics for patients in the ward and ICU. Two earlier investigations demonstrated that the likelihood of receiving antibiotic medication was affected by patients’ elevated CRP values [19, 20]. According to a report, 91% of doctors weigh the CRP level when administering antibiotics to COVID-19 patients [14]. CRP, a low-cost point of care, can assist in lowering doctors’ unjustified antibiotic prescribing. However, it is important to consider other factors outside bacterial infection that could increase CRP, an acute phase reactant. According to the national COVID-19 guide of the Ministry of Health in our nation, the existence of elevated CRP levels alone should not be a justification for initiating antibiotics. Instead, CRP values may rise as a result of the hyperinflammatory response in these individuals [11].

In a multicenter study conducted by Beovic et al., ceftriaxone/cefotaxime + macrolide and piperacillin/tazobactam were reported as the most commonly used antibiotics for the treatment of COVID-19 patients in the ward and ICU, respectively. However, only 46 physicians from Turkey participated in this study [9]. In a few studies involving hospitalized patients in our nation, respiratory quinolones were the most frequently prescribed antibiotics [5–10, 13]. Similarly in our study the most prescribed antibiotics were respiratory quinolones. In addition, the study’s responders claimed that they frequently prescribed certain types of antibiotics to COVID-19 patients with varied degrees of severity (outpatient, inpatient, and intensive care unit). The majority of doctors in the ward were found to choose respiratory quinolones and 2nd or 3rd generation cephalosporins as the first line treatment for COVID-19 patients. Patients with COVID-19 were apparently given respiratory quinolones and carbapenems in the intensive care unit. Respiratory quinolones were identified as the primary therapeutic option of choice for the large number of doctors even in the treatment of minor COVID-19 patients, although both national and international recommendations do not suggest the use of any antibiotic for the treatment of COVID-19 sickness [11, 15].

The study has both strengths and limitations. Because of the characteristics of a survey study, our work has limitations. All data are based on the individual’s notification. Selection bias and recall bias are the limitations of this study. Although participation from many cities of Turkey is present, it does not represent the entire country. Because we distributed the poll via social media, we were unable to determine the response rate. Second, the vast majority of respondents were pulmonologists. The study’s participation rate was particularly low among infectious disease. Nonetheless, our study provides important data on physicians’ antibiotic prescribing views for COVID-19 patients. This study is a large and widely attended study that includes data of healthcare-workers from all geographic regions of Turkey. We also attempted to discover practice variety by enrolling people from various venues and physician specialties.

In conclusion, we found that physicians in Turkey frequently use irrational antibiotic prescribing practices to treat COVID-19 patients, even those with mild disease. This may increase the likelihood of antibiotic-related side effects, antimicrobial resistance, and economic burden. Specific feasible interventions such as postspecialty repetitive training, are needed to encourage and maintain the judicious use of antibiotics.

### Electronic supplementary material

Below is the link to the electronic supplementary material.

Supplementary Material 1

## Data Availability

No datasets were generated or analysed during the current study.
