# Supplementary material for: Physicians’ irrational attitudes on the antibiotic prescribing for the treatment of COVID-19 in Turkey: A multicenter survey
Source: BMC Health Serv Res. 2024 May 21;24:650. doi: 10.1186/s12913-024-11110-z (PMC11110415; doi:10.1186/s12913-024-11110-z)
Supplement: Supplementary file 1 — Supplementary Material 1 [file 12913_2024_11110_MOESM1_ESM.docx]

**Physicians’ irrational attitudes on the antibiotic prescribing for the treatment of COVID 19 in Turkey: A Multicenter Survey**

This short survey, which will take you approx 3 minutes, is trying to help us identify the antibiotic prescribing attitudes of the physicians for treatment of COVID 19. Your personal data will not be shared.

Do you agree to answer our questionnaire?

| Yes |  |
| --- | --- |
| No |  |

**Q1** Please insert your age

|  |
| --- |

**Q2** please specify your gender

|  |
| --- |

**Q3** Please specify the city you work

|  |
| --- |

**Q4** Please specify your area of specialization

|  |
| --- |

**Q5** Please specify in which year of your profession you are

| 1-5 |  |
| --- | --- |
| 5-15 |  |
| 15-25 |  |
| >25 |  |

**Q6** Please specify your medical degree

|  |
| --- |

**Q7** Please specify the type of the hospital where the patient was applie

|  |
| --- |

**Q8** Did you treat any COVID-19 patients in the outpatient clinic since the first COVID-19 case was identified?

| Yes |  |
| --- | --- |
| No |  |

**Q9** What percentage of COVID-19 patients in the outpatient clinic do you prescribe antibiotics for?

| 0-20% |  |
| --- | --- |
| 20-40% |  |
| 40-60% |  |
| 60-80% |  |
| 80-100% |  |

**Q10** What is/are your most usual empirical choice(s) of antibiotics in patients with COVID-19 *in the outpatient*?

| Cephalosporins |  |
| --- | --- |
| Penicillin / amoxicillin |  |
| Macrolide |  |
| Fluoroquinolone |  |
| Combination |  |
| I do not routinely prescribe antibiotics to the outpatient  patients in the outpatient |  |

**Q11** Did you treat any COVID-19 patients in the inpatient clinic since the first COVID-19 case was identified?

| Yes |  |
| --- | --- |
| No |  |

**Q12** Do you make the decision to choose antibiotics for the hospitalized patient based on empirical or microbiological examination results?

| Empirical |  |
| --- | --- |
| Microbiological examination |  |

Q13 What percentage of COVID-19 patients in the inpatient clinic do you prescribe antibiotics for?

| 0-20% |  |
| --- | --- |
| 20-40% |  |
| 40-60% |  |
| 60-80% |  |
| 80-100% |  |

**Q14** What is/are your most usual empirical choice(s) of antibiotics in patients with COVID-19 *on the ward*?

| 2nd or 3rd generation Cephalosporins |  |
| --- | --- |
| Beta lactam+beta lactamase combination |  |
| Macrolide |  |
| Fluoroquinolone |  |
| Anti-pseudomonal beta-lactam |  |
| Carbapenems |  |
| Glycopeptides |  |

**Q15** Did you treat any COVID-19 patients in the ICU since the first COVID-19 case was identified?

| Yes |  |
| --- | --- |
| No |  |

**Q16** Do you make the choice of antibiotic for the patient in the intensive care unit based on empirical or microbiological examination results?

| Empirical |  |
| --- | --- |
| Microbiological examination |  |

**Q17** What percentage of COVID-19 patients in the ICU do you prescribe antibiotics for?

| 0-20% |  |
| --- | --- |
| 20-40% |  |
| 40-60% |  |
| 60-80% |  |
| 80-100% |  |

**Q18** What is/are your most usual empirical choice(s) of antibiotics in patients with COVID-19 *in intensive care unit?*

| 2nd or 3rd generation Cephalosporins |  |
| --- | --- |
| Beta lactam+beta lactamase combination |  |
| Macrolide |  |
| Fluoroquinolone |  |
| Anti-pseudomonal beta-lactam |  |
| Carbapenems |  |
| Glycopeptides |  |

**Q19** Which parameter(s) important for the decision to prescribe antibiotic treatment in patients with COVID-19?

| Fever |  |
| --- | --- |
| Cough |  |
| Sputum purulence |  |
| Dyspnea |  |
| Comorbidities |  |
| Mutated variant |  |
| Saturation |  |
| Vaccination status |  |
| Abnormal radiological findings |  |
| C- reactive protein |  |
| Procalcitonin |  |
| D-dimer |  |
| White-blood cell count |  |
| Neutrophil count |  |
| Lymphocyte count |  |
| Ferritine |  |
| Sputum microbiological analysis |  |
| Other |  |

**Q20** Have you ever prescribed combinations of antibiotics for COVID-19 Patient?

| Yes |  |
| --- | --- |
| No |  |

**Q21** Where do you prefer combinations antibiotic therapy?

| Outpatient |  |
| --- | --- |
| Ward |  |
| ICU |  |
